# Chronic Abdominal Syndrome Due to Nervous Compression. Study of 100 Cases and Proposed Diagnostic-Therapeutic Algorithm

**DOI:** 10.1007/s11605-015-2801-8

**Published:** 2015-03-24

**Authors:** Francisco Javier Pérez Lara, J. Quintero Quesada, J. A. Moreno Ramiro, R. Bustamante Toledo, A. Del Rey Moreno, H. Oliva Muñoz

**Affiliations:** 1 Surgery Service, Antequera Hospital, Avda Poeta Muñoz Rojas sn., 29200 Antequera, Málaga Spain; 2 Unit of Spinal Column, Traumatology Service, Antequera Hospital, Málaga, Spain; 3 Radiology Service, Antequera Hospital, Málaga, Spain; 4 Neurology Service, Carlos Haya Hospital, Málaga, Spain

**Keywords:** Chronic abdominal pain, Nervous compression, Digestive symptoms, Urologic symptoms, Gynaecologic symptoms

## Abstract

**Objective:**

In the medical literature, thoracic disc protrusion has traditionally been considered a rare occurrence. We hypothesise that the incidence of such protrusions and their abdominal symptoms is higher than is generally believed and that their presence may account for a significant proportion of chronic non-visceral abdominal pains. Accordingly, the present study was designed to identify and quantify the symptoms experienced by patients with thoracic disc protrusion and to assess the relative risk of these symptoms being presented, compared to the general population.

**Design:**

We conducted a cross-sectional study with a control group. The following comparison groups were analysed: case group, consisting of 100 patients diagnosed with thoracic disc protrusion in our hospital between February 2007 and October 2012, and control group consisting of 100 subjects from the general population, chosen at random. To compare the symptoms observed in each group, the following tests were applied to all study subjects: clinical examination, gastrointestinal-related quality of life (GIQLI) questionnaire and DN4 questionnaire. We also reviewed the subjects’ medical records for the previous 3 years.

**Results:**

The subjects in the case group had a significantly higher incidence of digestive-urologic symptoms, a poorer gastrointestinal quality of life and greater need of medical care than those in the control group. The differences were statistically significant for all the parameters studied. Almost all the case group subjects suffered chronic abdominal pain and/or digestive-urologic symptoms. We term this group of symptoms “chronic abdominal syndrome due to nervous compression”. Nevertheless, in most cases, no neurologic aetiology was suspected, and therefore the treatment given was ineffective. In view of the results obtained, we propose a diagnostic-therapeutic algorithm for such patients.

**Conclusion:**

Thoracic disc protrusion, as well as having a non-negligible incidence, is often associated with a digestive-urologic clinical syndrome, and this factor should be taken into account in all cases of chronic abdominal pain and other digestive-urologic symptoms when standard tests are negative, so that appropriate treatment may be given.

## Introduction

Chronic abdominal wall pain is often mislabelled as being visceral in origin and so inappropriate complementary tests are performed, unsatisfactory treatment is prescribed and unnecessary costs are incurred. The prevalence of this condition in everyday medical practice is unknown, but some researchers have estimated that it affects about 10 % of the patients presenting with chronic idiopathic pain at a gastroenterology consultation.^1^ Therefore, the early exclusion of a parietal cause would increase diagnostic accuracy for these patients.

The dorsal roots facilitate abdominal cutaneous innervation and visceral innervation of the colon, bladder and pelvic structures. Therefore, any dysfunction can produce abdominal pain and gastrointestinal symptoms (diarrhoea, constipation, increased peristalsis, abdominal pain, tenesmus, etc.), urologic complaints (such as dysuria or polyaquiuria) and pain at diverse metameric levels (in the abdomen, pubis, groin, testicular area, trochanter, etc.).

It has traditionally been considered that the incidence of thoracic disc protrusion (TDP) is very low, amounting to less than 1 % of all disc protrusions.^2^ However, in recent years, new diagnostic imaging methods have revealed otherwise; in fact, the incidence of TDP is between 11 and 37 %.^3^^–^ 7 Similar findings have been reported from necropsy studies, according to which the incidence may be as high as 10 % of the population.^8^

Despite this high incidence, only 0.5–0.8 % of cases are considered symptomatic.^9^^,^ 10 On the other hand, it should also be borne in mind that in most cases, only neurologic symptoms are considered (no further examination is made as to whether the patient has abdominal pain or digestive-urologic symptoms), and so when all of these symptoms are viewed jointly, the percentage of symptomatic patients may be much higher.

Therefore, the problem could be addressed like this: neither the orthopaedic surgeon nor the neurologist see patients with chronic abdominal pain; on the other hand, the gastroenterologist, the gastrointestinal surgeon, the gynaecologist and the urologist do see patients with chronic abdominal pain, but they do not usually suspect the fundamental cause to be located in the spine and therefore do not ask for magnetic resonance imaging (MRI) of this area. In consequence, we are faced with a pathology of a neurologic-traumatologic nature but with gastrointestinal-urologic-gynaecologic symptoms, which makes diagnosis very difficult. As a result, to date, this body of symptoms has been considered to constitute a pathology with a low incidence. However, we believe that what is really infrequent is the diagnosis, not the condition.

Given these premises, in two recent papers^11^^,^ 12, we published the results of a two-phase study in which a spinal MRI was performed on all patients attending our clinic with chronic abdominal pain and symptoms (with criteria of parietal pain, after the presence of an organic pathology had been rejected). Of these patients 63.82 % (30 of 47) were found to have one or more thoracic disc protrusions. In the above papers, we described the symptoms of these patients and the results obtained after specific treatment was prescribed for neuropathic pain.

Taking into account the above results, the aim of the present study was to examine the syndrome complex presented by 100 patients with TDP, to assess whether they are being properly diagnosed and treated, to compare these patients with a control group and, finally, to propose a diagnostic and therapeutic algorithm for this type of patient.

## Material and Method

We conducted a cross-sectional study with a control group. Thus, two groups of 100 subjects were compared: group 1 (cases) consisted of 100 patients diagnosed with TDP between February 2007 and October 2012 in our hospital and group 2 (controls) consisted of 100 patients randomly selected from the general population (that of the hospital district). Both groups had similar characteristics in terms of sex (male/female 55/45 vs. 54/46, *p* = 1) and age (52.48 vs. 50.15 years, *p* = 0.316).

The study was approved by the research and ethics committee of our hospital. The subjects were informed about the aims and procedures of the study, and those who agreed to participate (cases: 100 of 145; controls: 100 of 135) signed an informed consent form and underwent a clinical test (Annex 1) to evaluate symptoms, diagnosis and treatment results. They also completed the gastrointestinal-related quality of life (GIQLI) questionnaire, either in the hospital or sending it later, by post. Subjects with abdominal pain were also given a specific questionnaire (DN4) to assess whether the pain was neuropathic in origin.

The GIQLI was presented in 1994 by Eypasch et al.^13^^,^ 14 and it was translated into Spanish and validated in 2000.^15^ This questionnaire is a mixed type, between generic and specific, since it is used both to evaluate parameters related to the overall quality of life and to examine those which relate specifically to the upper and lower gastrointestinal tract. It has become increasingly popular in recent years, since it is simple to administer and can be completed quickly and easily by the subject, without expert assistance. It consists of 36 questions organised into four dimensions: gastrointestinal symptoms, emotional role (degree of tolerance to daily stress, depression, stress, nervousness, fear, satisfaction with life and level of frustration), physical status (tiredness, fatigue, illness, insomnia, abnormal changes in physical appearance, vitality, stamina and fitness) and social role (ability to perform daily living or recreational activities, changes in relationships with friends and family, and sexual quality of life). Both the final result and each of the dimensions are scored from 0 to 4 (with 0 being the worst and 4 being the best score).

We used the DN4 questionnaire^16^ (Annex 2) to evaluate the possible neuropathic aetiology of the abdominal pain. This questionnaire consists of ten items grouped into four sections. The first seven items are related to the nature of the pain (burning, painful cold, electric shocks) and its association with abnormal sensations (tingling, pins and needles, numbness, itching). The other three items are related to the neurologic examination in the area of pain (hypoesthesia to touch, hypoesthesia to pricking, tactile allodynia). Each positive item is scored 1 point and each negative item is scored 0. The total score is calculated by summing the ten items, and the cutoff value for the diagnosis of neuropathic pain is a total score higher than 3.

The DN4 questionnaire was adapted into Spanish and its psychometric properties (reliability and validity) were evaluated by Pérez et al. 17 The questionnaire has a sensitivity of 87 % and a specificity of 84 %.

Finally, we reviewed the clinical record, for the previous 3 years, of the subjects in each group, including treatment at the hospital’s emergency department, consultations with specialists, complementary tests performed and hospital admissions.

These tests and the review of medical records enabled us to evaluate the following parameters for the patients with TDP:

- Chronic pain, and its intensity, in the abdomen, back, genitals and/or lower limbs (clinical test)
- Percentage of abdominal pain of a neuropathic nature (DN4 questionnaire)
- Digestive-urologic symptomatology (clinical test)
- Gastrointestinal quality of life (GIQLI questionnaire)
- Need for emergency department treatment, consultation with a specialist or hospital admission during the last 3 years (medical history)
- Percentage of patients whose symptoms were attributed to TDP (clinical test)
- Medical treatment conducted (clinical test)
- Outcome of medical treatment conducted (clinical test)

We compared the results for these patients (cases) with those for the randomly selected control group, to assess the deterioration of gastrointestinal quality of life and the relative risk of these patients presenting with the symptoms described, with respect to the general population.

From the results obtained, and our experience with these patients in previous studies, we will describe the clinical picture presented and the inclusion criteria applied. We will also review recent literature in this respect and propose an algorithm for diagnostic and treatment action.

### Statistical Study

In a descriptive analysis of the study variables, the values of the continuous variables are presented as the mean and the corresponding standard deviation. Categoric variables are presented as absolute and relative frequencies. The results are shown stratified by groups (case/control).

To test whether the data—the observed differences in the frequencies of the variables of interest—are statistically significant, Fisher’s test was used to assess the qualitative variables. In addition, the prevalence ratio was calculated, at a 95 % confidence interval.

To analyse the differences between the continuous quantitative variables in two independent groups, Student’s *t* test was applied for two independent samples. This was done assuming the normal distribution of the variables in each group, which was confirmed by the Shapiro-Wilk test. When non-normality was observed, the Mann-Whitney non-parametric test was used.

The statistical analysis was performed by the FIMABIS AMEC Unit, Málaga (Spain), with software R project version 3.0.3.

## Results

**Fig. 1:**
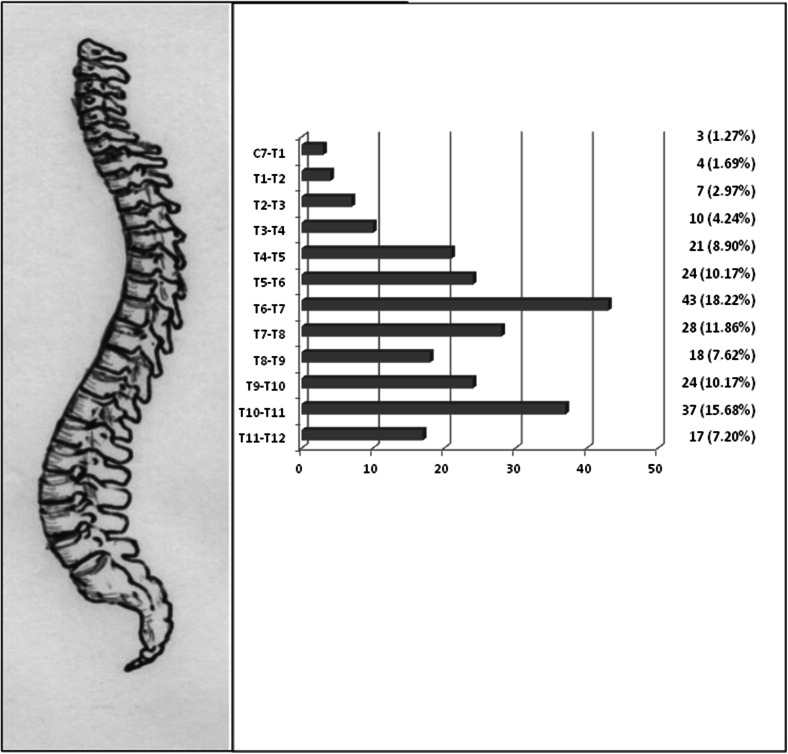
Location of disc herniations (case group)

The 100 subjects in the case group presented a total of 236 TDP. In 77 % of these, the subarachnoid space was occupied, and in 8.89 %, there was contact with the bone, but only in 1.69 % was there injury to the nerve root. Of the protrusions, 70.76 % were below the T7 level (Fig. 1). The disc protrusion was generalised in 10.59 % of cases, central in 65.25 %, right paracentral in 7.2 % and left paracentral in 16.95 %.

Of the subjects with TDP, 77 % had abdominal pain, and of these, 16.88 % reported having had an operation to relieve the pain (five cholecystectomies, three umbilical hernia repairs, one inguinal hernia repair, one suprarenalectomy, one appendectomy, one hysterectomy, one oophorectomy), but in all cases, the pain persisted after surgery. In 50.64 % of cases, the patients commented that the pain occurred when certain postures were adopted, 49.35 % related it to physical effort and 12.99 % referred to a prior traumatic injury.

Of the patients in the case group, 95 % presented one or more digestive-urologic symptoms, but only 3 % reported having had a diagnosis of TDP as the cause of their symptoms. With respect to the medical treatment prescribed for their abdominal pain, 25 % had not been given any treatment, 68 % had been treated with NSAIDs and only 7 % had received treatment for neuropathic pain (3 % stage 1, 2 % stage 2, 1 % stage 3 and 1 % stage 4).

In response to the question, “What changes have you noticed after the treatment prescribed by your doctor?”, 8 % of the patients stated that the pain had disappeared, 12 % had obtained a significant improvement, 21 % had obtained a moderate improvement, 31 % had obtained a slight improvement and 28 % had obtained no improvement or a worsening of the pain.

On comparing the two groups, we observed that both the pain and the digestive-urologic symptoms were significantly more frequent in the case group, with statistical significance in all items (Table 1).

**Table 1:**
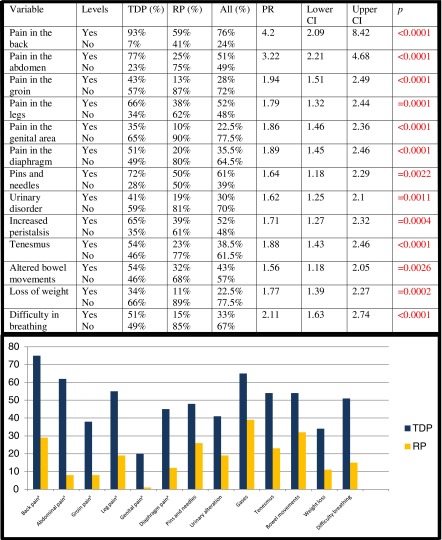
Comparison of symptoms in cases/controls

^a^Only symptoms with intensity ≥5/10 are included

*TDP* thoracic disc protrusion, *RP* random population, *PR* prevalence ratio, *CI* confidence interval

The subjects in the control group who had abdominal pain reported it to be most frequently located in the central areas, especially periumbilical locations (zone 5) (32 %), while in the case group, the abdominal pain was most often located in lateral and lower areas, especially in zone 7 (in the right iliac fossa) (33 %).

Both the duration and the intensity of the pain (Table 2) were greater in the case group, with statistical significance in all locations.

**Table 2:**
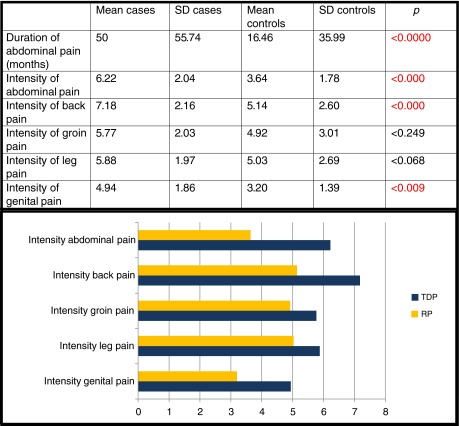
Comparison of cases/controls: duration of pain and pain intensity in different areas

*SD* standard deviation, *TDP* thoracic disc protrusion, *RP* random population

Visits to the hospital’s emergency department, consultation with specialists and hospital admissions in the previous 3 years were all more numerous in the case group, with statistical significance in the latter two cases (Table 3).

**Table 3:**
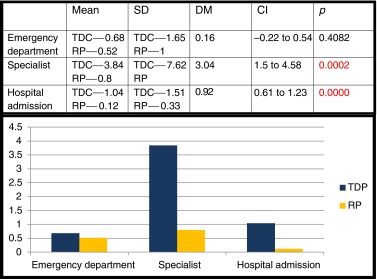
Comparison of cases/controls: medical attention required in the last 3 years

*SD* standard deviation, *DM* difference of the means, *CI* confidence interval, *TDC* thoracic disc protrusion, *RP* random population

The number of complementary tests performed in the previous 3 years (Table 4) was clearly higher in the case group, with statistical significance in all items.

**Table 4:**
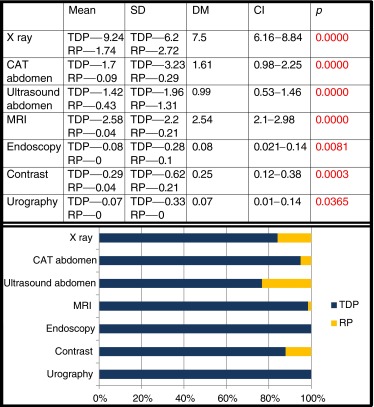
Comparison of cases/controls: complementary tests in the last 3 years

*SD* standard deviation, *DM* difference of the means, *CI* confidence interval, *TDC* thoracic disc protrusion, *RP* random population

The gastrointestinal quality of life was significantly lower in the case group, with statistical significance in all items (Table 5).

**Table 5:**
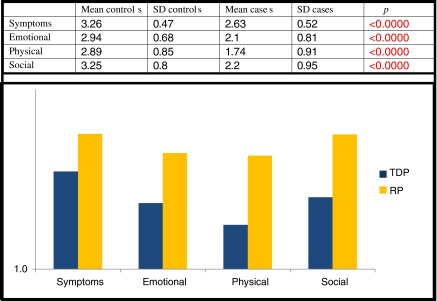
Comparison of cases/controls: GIQLI questionnaire

*SD* standard deviation, *TDC* thoracic disc protrusion, *RP* random population

Finally, 84.42 % of the subjects in the case group with abdominal pain (*n* = 77) had a positive result in the DN4 test, with an average score of 4.27 (SD 2.1), However, among the subjects with abdominal pain in the control group (*n* = 25), only 8 % had a positive result in this test. They had an average score of 1 (SD 1.08), and the differences were statistically significant with respect to positive test results (*p* = 0.000) and average score (*p* < 0.001).

## Discussion

Chronic abdominal pain, together with symptoms of gastrointestinal disturbances (of a non-organic nature), is commonly encountered in current clinical practice and is normally classed as idiopathic gastroparesis or functional disorder (irritable bowel syndrome or functional dyspepsia, among others).^18^

This problem continues to present a challenge to all currently known methods of diagnosis and treatment.^19^ Patients usually consult various doctors and undergo multiple complementary tests, but in many cases, these fail to identify the cause of the problem.

In 30 % of all patients with chronic abdominal pain, it is located in the abdominal wall.^20^ The first reference to non-visceral abdominal pain was made by Cyriax,^21^ who was convinced that among a large number of patients, their abdominal pain was not attributable to visceral causes.

However, it was Carnett, an English obstetrician, who first attributed this pain to the structures of the abdominal wall, and in 1926, he described the sign that bears his name, and which remains in use today in clinical examination.^22^ This sign has a sensitivity of 78–85 % and a specificity of 88–97 %.^23^ In this procedure, the patient is placed in a supine position and the abdomen is palpated to identify the area of greatest sensitivity to pain. The patient is then asked to raise his head and shoulders to facilitate the contraction of the abdominal muscles. If the pain increases (or remains unchanged), its cause is most likely in the abdominal wall and therefore the Carnett test result is positive.^24^

The patient is not usually able to interpret the degree of superficiality of the pain, and therefore the physician must take into account the clinical history and perform a careful examination in order to determine the metameric source of the pain. An algorithm for evaluating chronic abdominal pain, using Carnett’s sign and the infiltration of the trigger points (if the pain decreases when the point of maximum pain is infiltrated, the sign is positive), has been suggested by Gallegos and Hobsley^25^ and was used by Greenbaum et al.^24^

In a study conducted by Constanza et al. of physicians treating patients with chronic abdominal pain, only 4 % of respondents considered pain originating in the abdominal wall as an initial diagnostic possibility.^26^ This finding coincides with our own results, in which in only 3 % of patients was the pain attributed to a disc pathology.

Gómez Rodríguez et al.^27^ reported that when abdominal pain of unknown aetiology is persistent, this leads to multiple examinations—both time- and labour-intensive—being conducted, and if a small anomaly is found, it can sometimes lead to unnecessary surgery being performed, especially in gynaecological medicine. This is corroborated by our own results, according to which the patients in the case groups underwent a large number of additional tests, specialist consultations and hospital admissions, in comparison with the general population, and 16.88 % of these patients were operated for pathologies unrelated with the metameres of the abdominal wall, and unsuccessfully in every case.

Since symptomatic TDP was first described by Key in 1938, this uncommon pathology has posed a challenge to spinal surgeons.^28^ Both diagnosis and treatment are controversial due to the low prevalence described, the wide variety of clinical presentations and conflicting definitions of discal symptoms.^29^

Visceral and somatic afferent fibres in the dorsal columns, spinothalamic and spinocerebellar tracts and the dorsal and ventral horns have been observed at different levels of the spine.^30^^–^ 32 The irritation of these tracts, and the fact of their close association with the dorsal grey column of the spinal cord, can cause pain and discomfort.^31^ Rohde and Kang proposed that compression of the cord at the site of visceral afferent fibres can lead to inflammation and hyperexcitability of the visceral neurons.^30^ This might interfere with the descending inhibitory fibres that modulate noxious input and thus provoke atypical presentations of TDP. This would account for the clinical variability found in our study (cases with bilateral pain, cases with an evident visceral association and cases with lesions higher than the T7 level but presenting abdominal pain).

In agreement with our results, Arce and Dohrmann^5^ reported that 75 % of TDP occur below T8, 3 % between T1 and T2 and less than 1 % between T2 and T3. As regards the area of the disc that is affected, central protrusions are the most common.^33^ This condition is observed more frequently in middle-aged and older patients, and there are no significant differences with respect to gender.^34^^,^ 35 Although disc degeneration is the primary factor, trauma is also of considerable importance, being recorded in up to 25 % of cases.^5^^,^ 33 Nevertheless, in our study, only 12 % of the patients in the case group had a trauma event in their clinical history. Genetic factors have also been suspected as a possible cause.^36^

From our review of the medical literature in this respect, we conclude that in most cases, symptomatic TDP has a mild to moderate clinical impact, producing thoracic or abdominal pain, sensory disturbances^37^^,^ 38 and, less frequently, myelopathy and weakness of the lower limbs.^39^ Other gastrointestinal, urologic and cardiopulmonary symptoms have also been described.^40^^,^ 41 This varied set of clinical presentations cannot be correlated with the location of the hernia, and so patients are often misdiagnosed by their primary care physicians, which gives rise to extensive and costly tests being performed.^42^ If the patient is referred to a specialist (normally a gastroenterologist, gastrointestinal surgeon, urologist or gynaecologist), a disorder of a neurologic nature is unlikely to be suspected. In consequence, TDP has traditionally been considered a very uncommon disease (one case per million inhabitants per year^2^).

However, recent necropsy studies of the spine have suggested that the prevalence may be as high as 10 %.^8^ Moreover, new imaging techniques have detected TDP in 11–37 % of the population^3^^–^ 7 and 39 % of cases have more than one herniation.^6^

This strongly suggests that we are facing a disease with a high incidence among the population, and therefore many patients are currently undiagnosed. For example, in the catchment area for our hospital, with a population of about 100,000, according to the above studies, there must be between 10,000 and 37,000 people with TDP. The question then arises: what percentage of these people present well-developed abdominal pain with gastrointestinal, gynaecologic or urologic symptoms and yet are undiagnosed and therefore not being treated properly? To put it another way, how many patients with chronic abdominal pain and symptoms, in cases in which organic pathology has been excluded, and who are normally classified as suffering irritable bowel syndrome or a functional pathology, might actually be suffering a thoracic spine pathology, which is the true cause of their condition? Answers to these questions can only be properly obtained by carrying out a prospective multicentre study with a large number of patients. Contrary to the findings of previous research in this field, our study shows that patients with TDP very often suffer abdominal pain (77 %), accompanied by digestive-urologic symptoms (95 %) and a significant deterioration in their gastrointestinal quality of life. It seems, therefore, that a significant number of patients could benefit from the diagnostic and therapeutic algorithm proposed in this study.

When we asked our patients with TDP about the diagnosis they had been given, only 3 % mentioned neuropathic pain. In view of the long-standing nature of the condition (mean duration 50 months) and the fact that most of these patients had not received a definitive diagnosis, there remains an important knowledge gap to be filled. In addition, it should be taken into account that 25 % of these patients were not treated and that 68 % had had a treatment that did not correspond to the scale of neuropathic pain. Furthermore, only 20 % reported being pain-free or having achieved a significant improvement after treatment. It must be concluded that a large portion of these patients were inadequately treated; no aetiological diagnosis of nervous compression was made, and therefore the outcome was unsatisfactory. In our previous two studies, when patients were diagnosed with disc pathology as being responsible for the pain, this was treated by considering the neuropathic pain scale, and the pain was reduced or significantly alleviated in 61.11 and 53.33 % of cases, respectively.

Taking into account the data obtained in the present study and the findings of previous research by our group, we term the symptoms presented by these patients “chronic abdominal syndrome due to nervous compression” (CASNC). We define this syndrome as one in which there is chronic abdominal pain, often accompanied by back pain and digestive-urologic symptoms, with no apparent cause of an organic nature being revealed by the complementary tests; moreover, TDP has been observed and at least one of the following criteria is met:

- Carnett’s sign +
- Trigger point infiltration +
- DN4 questionnaire +

**Fig. 2:**
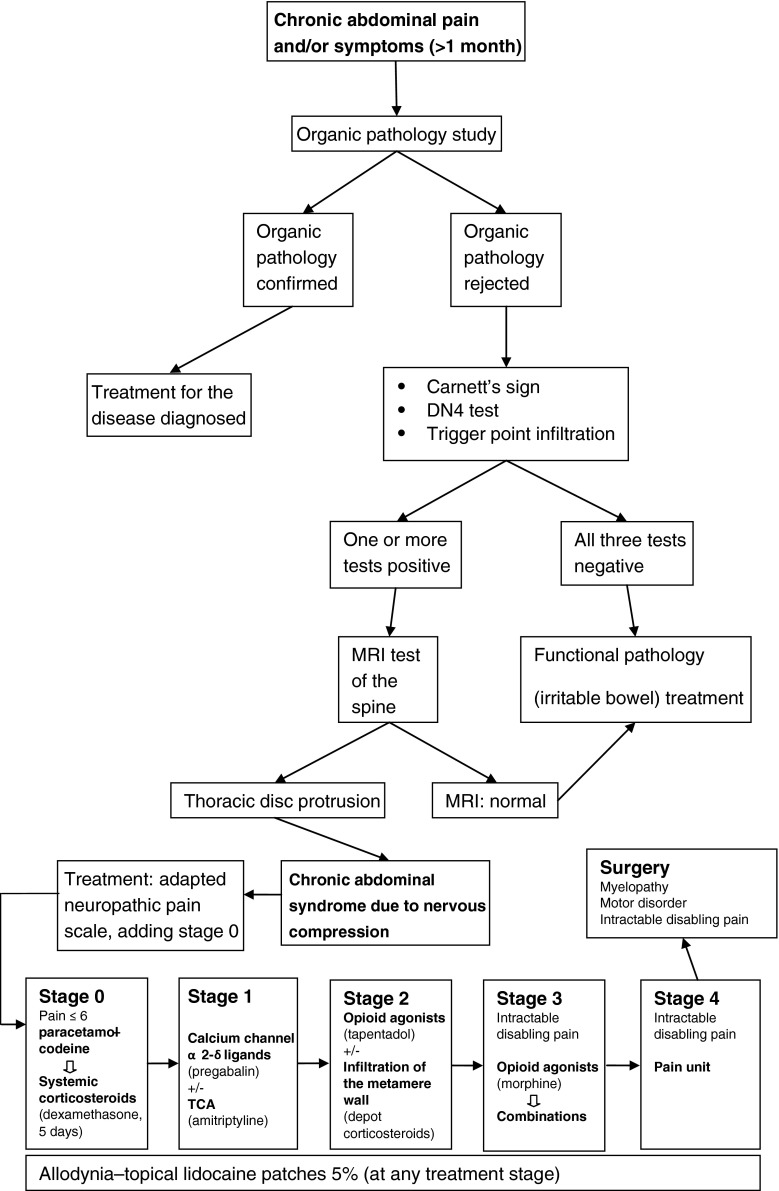
Diagnostic and therapeutic algorithm

The ideal treatment for neuropathic pain would be to address and resolve its cause. However, this is often not possible, and when it is undertaken, the pain may only be alleviated in part or even not at all. In such cases, excepting the peculiarities of very specific types of pain, the pharmacologic treatment of neuropathic pain is carried out in stages, using different pharmacologic groups. In our opinion, this is the approach that should be followed in patients diagnosed with CASNC. However, there is no consensus as to the drugs that should be employed at each stage, and CASNC patients form a highly specific group. Therefore, we decided to adapt the pain scale for the treatment of CASNC taking into account our own previous studies and the latest guidelines for the treatment of neuropathic pain (published by the International Association for the Study of Pain, the Neuropathic Pain Special Interest Group, the European Federation of Neurological Societies and the Canadian Pain Society), together with recent studies in this respect.^43^^–^ 55 Finally, taking into account these considerations, we propose a diagnostic and therapeutic algorithm (Fig. 2) for all patients with TDP and chronic abdominal pain or gastrointestinal-urologic symptoms and for whom conventional diagnostic methods have ruled out the presence of an organic pathology.

## Conclusion

In summary, both TDP and its symptoms are much more common than has been thought. The majority of these patients present a long-standing clinical situation, with pain in the back (90 %), abdomen (77 %) or other locations (pubis 43 %, genital area 35 %, lower limbs 66 %), always at a considerable level of intensity and frequently accompanied by digestive-urologic symptoms (95 %). These symptoms provoke a significant deterioration in the gastrointestinal quality of life and lead patients to be offered a large number of complementary tests, consultations with specialists and even hospital admission. Nevertheless, after all these studies, the patients’ condition is usually summarised as being of a functional nature, and so inadequate treatment is provided and the results obtained are unsatisfactory.

As treatment strategies vary according to whether the pain is nociceptive or neuropathic, it is important to identify the neuropathic component, even when the pain is heterogeneous. Therefore, the possible presence of CASNC should be considered in all cases of chronic abdominal pain and symptoms with negative test results, so that appropriate treatment may be given.

## Figures and Tables

**Table 6:**
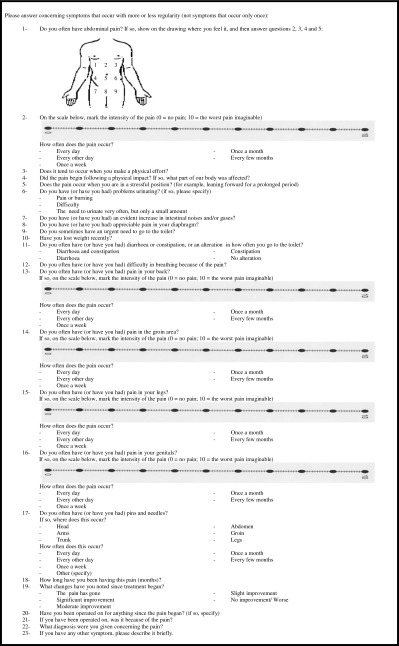
Clinical test

## Annex 1

ᅟ

## Annex 2

ᅟg

**Table 7:** DN4 questionnaire

Does the pain have one of more of the following characteristics?		
Burning	YES 1 point	NO 0 point
Painful cold	YES 1 point	NO 0 point
Electric shocks	YES 1 point	NO 0 point
Is the pain associated with one or more of the following symptoms in the same area?		
Tingling	YES 1 point	NO 0 point
Pins and needles	YES 1 point	NO 0 point
Numbness	YES 1 point	NO 0 point
Itching	YES 1 point	NO 0 point
Is the pain located in an area where the physical examination may reveal one or more of the following characteristics?		
Touch Hypoaesthesia	YES 1 point	NO 0 point
Pricking Hypoaesthesia	YES 1 point	NO 0 point
In the painful area, can the pain be caused or increased by:		
Brushing	YES 1 point	NO 0 point
